# Roles of NOTCH1 as a Therapeutic Target and a Biomarker for Lung Cancer: Controversies and Perspectives

**DOI:** 10.1155/2015/520590

**Published:** 2015-09-27

**Authors:** Lixia Guo, Ting Zhang, Ying Xiong, Yanan Yang

**Affiliations:** ^1^Thoracic Disease Research Unit, Division of Pulmonary and Critical Care Medicine, Department of Internal Medicine, College of Medicine, Mayo Clinic, Rochester, MN 55905, USA; ^2^School of Arts and Science, University of Pennsylvania, Philadelphia, PA 19104, USA; ^3^Department of Biochemistry and Molecular Biology, College of Medicine, Mayo Clinic, Rochester, MN 55905, USA; ^4^Developmental Therapeutics and Cell Biology Programs, Mayo Clinic Cancer Center, Mayo Clinic, Rochester, MN 55905, USA

## Abstract

Lung cancer is one of the most common types of human malignancies and the leading cause of cancer-related death. Patients with surgically resectable early stage lung cancer are more likely curable, but currently only a small population of patients can be diagnosed at such a stage, partly due to our incomplete understanding of the biology of lung cancer and the lack of diagnostic and prognostic biomarkers. Recent studies have shown that NOTCH1 is a critical regulator of human carcinogenesis and has been implicated in multiple steps of cancer development and progression. Herein, we review recent findings about the role of NOTCH1 in lung cancer and discuss its potential usefulness as both a therapeutic target and a biomarker for lung cancer.

## 1. Introduction

*Current Status of Lung Cancer*. Lung cancer is very difficult to be detected and treated early and has remained one of the major life-threatening malignancies worldwide. It is estimated that lung cancer is diagnosed in about 1.8 million patients and causes more than 1.5 million deaths each year [1, 2]. Partly due to a number of increasing risk factors, such as smoking and environmental pollution, the incidence of lung cancer has significantly increased in the past decades in many developing countries, for instance, China [2, 3]. In Western countries, lung cancer is also the leading cause of cancer death in both men and women. According to the American Cancer Society (http://www.cancer.org/), more than 150,000 patients will die from lung cancer each year in the United States alone. Despite our continuous technical improvements, the overall five-year survival rate of lung cancer patients only moderately increased in the past years and remains as low as about 15% (http://www.cancer.org/).

Non-small cell lung cancer (NSCLC) and small cell lung cancer (SCLC) are the two major lung cancer subtypes that account for about 80% and 10–20% of the total cases, respectively. Patients with surgically resectable early stage lung cancer (mostly stage I and stage II) have more treatment options and better overall prognosis. For instance, the five-year survival rate of stage I NSCLC is about 50%, much higher than that of stage IV NSCLC, which is less than 5% according to the National Cancer Institute (http://www.cancer.org/). However, only 10–20% of the patients can be diagnosed at such an early stage. At the time of diagnosis, most patients have developed advanced lung cancer that associates with metastasis, namely, the spread of lung cancer cells to distant sites (most frequently to the lymph nodes, the liver, the brain, the bones, and the adrenal glands). As there is no clinically effective therapy available for treatment or prevention of metastasis, it has remained the primary cause of lung cancer death. Thus, the identification of novel molecular targets and biomarkers for early detection and better treatment will significantly improve our capacity to manage this deadly disease.

**Figure 1:**
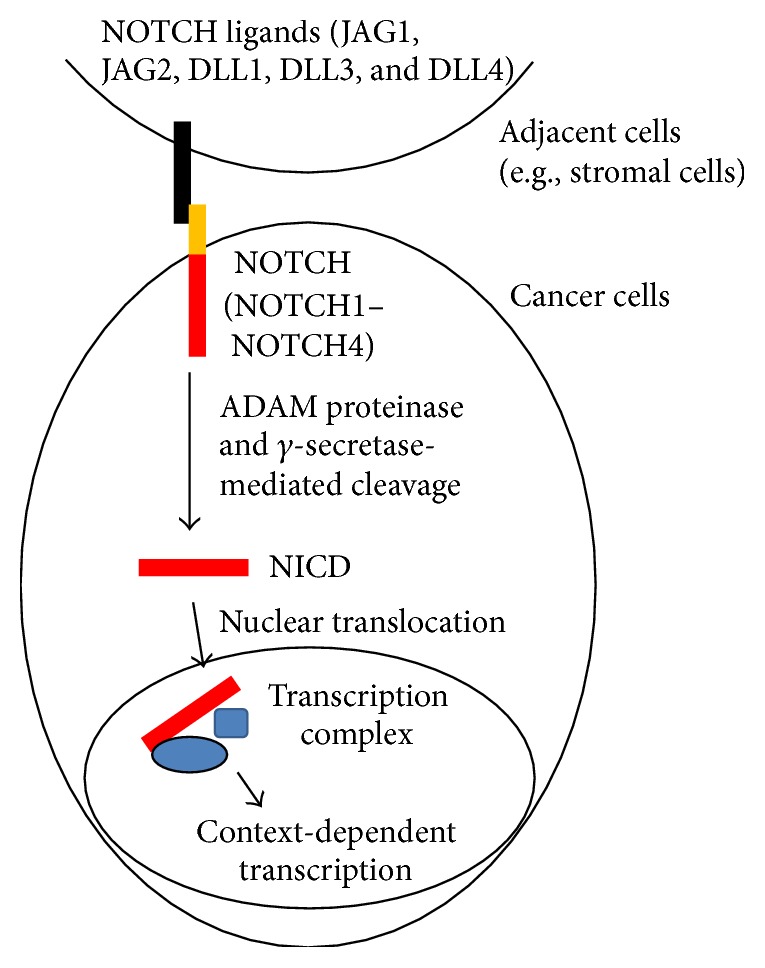
Schematic for NOTCH signaling in cancer cells. Upon the binding of NOTCH ligands (JAG1, JAG2, DLL1, DLL3, and DLL4) on adjacent cells, such as stromal cells or other cancer cells, NOTCH (NOTCH1–NOTCH4) on cancer cells can be activated through proteolytic cleavage (by ADAM proteinase and *γ*-secretase) to release its intracellular domain (NICD), which in turn translocates to the nucleus, where it forms transcription complexes with transcription cofactors (the blue shapes) to regulate cell-context-dependent transcription.

*NOTCH1 Signaling in Cancer Cells*. NOTCH1 belongs to the NOTCH family of transmembrane proteins that consist of four members (NOTCH1, NOTCH2, NOTCH3, and NOTCH4) and serve as receptors for NOTCH ligands, which are also membrane-bound proteins [4, 5]. As such, the interaction of NOTCH with NOTCH ligands on the surface of cancer cells and their adjacent cells (e.g., stromal cells or other cancer cells) plays a critical role in the cell contact-dependent intercellular communications (Figure 1). At least two classes of NOTCH ligands, including the Jagged proteins (JAG1 and JAG2) and Delta-like proteins (DLL1, DLL3, and DLL4), have been identified [4–6]. Upon the binding with NOTCH ligands, NOTCH1 will be cleaved by several proteases, including *γ*-secretases, to release its intracellular domain (NICD1), which can translocate to the nucleus and act as a transcription cofactor to regulate target gene expression in a cell-context-dependent manner (Figure 1; reviewed in [4–6]). Recent studies have shown that NOTCH1 is implicated in carcinogenesis in a variety of human malignancies by regulating many basic processes essential for cancer development and progression, including the cell growth, survival, apoptosis, migration, and invasion [6–9], suggesting a potential role for NOTCH1 as both a therapeutic target and a biomarker for early detection of cancer. In lung cancer, previous studies have shown that NOTCH1 acts as a driver of lung tumor initiation, growth, and metastasis. However, completely opposite findings were also reported, suggesting that the role of NOTCH1 in lung cancer is highly context-dependent and may be associated with disease subtypes or specific genetic changes.

## 2. Role of NOTCH1 in Lung Cancer Growth

### 2.1. A Putative Tumor Suppressor Role for NOTCH1 in SCLC Cells

Several studies using SCLC cell lines have suggested a tumor suppressor role for NOTCH1 (Table 1). For instance, stable expression of an active form of NOTCH1 (NICD1) in H446, H69, and H1688 cells inhibited cell proliferation and decreased the expression of neuroendocrine markers, such as CgA (chromogranin A), NSE (neuron-specific enolase), CGRP (calcitonin gene-related peptide), SYP (synaptophysin), and GRP (gastrin-releasing peptide) [10, 11]. Consistently, knockdown of NOTCH1 in H69AR and SBC-3 cells promoted both the expression of these markers and cell proliferation [11]. In addition, adenoviral expression of NOTCH1 in DMS53 and NCI-H209 SCLC cells was shown to promote growth inhibition by inducing cell cycle arrest at G1 phase, to increase the expression of cell cycle inhibitors p21^WAF1/CIP1^ and p27^KIP1^, and to reduce the expression of human achaete-scute homologue-1 (hASH1), a basic-helix-loop-helix (bHLH) transcription factor and a putative oncogene that can cooperate with SV40 T antigen to drive lung carcinogenesis [12, 13]. Thus, targeting NOTCH1 may promote SCLC cell growth.

### 2.2. Distinct Roles of NOTCH1 in NSCLC Cell Growth

In NSCLC cells, distinct roles of NOTCH1 in the regulation of cell growth have been reported (Table 1). In support of an oncogenic role for NOTCH1, it was reported that the knockdown of NOTCH1 by expressing its short hairpin RNAs (shRNAs) in NOTCH1-positive NSCLC cell lines, including H292, H358, H1650, H1975, and H2170 cells, significantly inhibited their anchorage independent growth in soft agar [14]. Interestingly, the expression of NICD1 in H292 cells specifically increased their proliferation and soft agar colony growth in the presence of EGF (epidermal growth factor), but not in the absence of EGF, suggesting that the activation of EGFR (epidermal growth factor receptor) may be essential for NICD1-dependent malignant transformation and tumor growth [14]. Under the condition of hypoxia, NOTCH1 can be activated by HIF1*α* (hypoxia-inducible factor 1-*α*) in lung adenocarcinoma cells (e.g., A549 cells), and the activated NOTCH1 suppressed the expression of PTEN (phosphatase and tensin homolog) and increased the expression of IGF-1R (insulin growth factor 1 receptor) to activate AKT (also known as protein kinase B), which in turn inhibited cell death and promoted cell growth [15]. The same group also reported that treatment with *γ*-secretase inhibitor, MRK-003, specifically induced lung adenocarcinoma cell apoptosis (A549 and H1755 cells) under the condition of hypoxia (1% O_2_), but not normoxia (21% O_2_) [16], suggesting that the biological role of NOTCH1 in lung adenocarcinoma cells may be dependent on the concentration of oxygen and that the pharmacological inhibition of NOTCH1 may be useful for treating hypoxia-induced and NOTCH1-driven lung tumor cell growth.

In a sharp contrast to the above results, others also presented evidence that supports a tumor suppressor role for NOTCH1 in NSCLC cells (Table 1). For instance, forced expression of NICD1 in A549 lung adenocarcinoma cells was sufficient to induce cell cycle arrest at G1 phase and to inhibit cell growth (in both two-dimensional and soft agar cultures) and tumor xenograft in nude mice [17]. Consistently, treatment of A549 cells with Z-Isochaihulactone, a key component of the crude acetone extract of* Bupleurum scorzonerifolium* (BS-AE), induced the expression of NICD1, arrested the cell cycle at G2/M phase, and inhibited cell proliferation [18]. Moreover, in endothelial and tumor cells' coculture model, it was shown that the expression of a NOTCH ligand DLL4 (Delta-like ligand 4) in HUVEC endothelial cells increased NOTCH1 and suppressed the proliferation of cocultured A549 and H460 NSCLC cells [19, 20]. Notably, this work also showed that the expression of DLL4 or NOTCH1 promoted the expression of PTEN [19], a tumor suppressor gene that antagonizes PI3K (phosphatidylinositol 3-kinase) signaling by dephosphorylating PIP3 (phosphatidylinositol (3,4,5)-trisphosphate) [21]. This result contradicts a previous finding from others [15], suggesting that the regulation of PTEN and PI3K signaling by NOTCH1 in NSCLC cells may highly depend on cellular contexts. Such distinct effect of the NOTCH1 signaling on PTEN expression might partly explain why NOTCH1 can exert completely opposite growth regulating functions. On the other hand, recent work from another group showed that the DLL4-mediated NOTCH1 activation in endothelial cells derived from LLC (Lewis lung carcinoma cells) tumor xenografts was also involved in the negative regulation of tumor angiogenesis and growth [22], suggesting that NOTCH1 signaling in both tumor cells and tumor microenvironment may cooperatively suppress tumor growth.

### 2.3. Lessons from Mouse Models of Lung Cancer

The biologic basis for the distinct actions of NOTCH1 in regulating lung cancer cell growth is not well understood. As mentioned above, there is a possibility that the role of NOTCH1 may be dependent on the cellular contexts. In support of such an idea, studies from several groups have shown that NOTCH1 is required for lung tumorigenesis in genetically engineered mouse models of lung adenocarcinoma that commonly express mutant* KRAS* (Table 1). For instance, in the* LSL-Kras* ^*G12D*^;* Notch1* ^*lox/lox*^ mice, conditional knockout of* NOTCH1* in the lung by intranasal instillation of adenovirus particles of the Cre recombinase, which also concomitantly induced the expression of* Kras* ^*G12D*^ in the lung, significantly suppressed the formation of lung adenocarcinomas [23, 24]. It was further shown that NOTCH1 may promote the mutant* KRAS*-dependent lung tumor formation in these mouse models by destabilizing p53 to suppress p53-mediated apoptosis [23] or by repressing DUSP1 (dual specificity protein phosphatase 1), a phosphatase that dephosphorylates and inactivates the growth-stimulating MAP kinases ERK1/2 [24]. On the contrary, inactivation of NOTCH1 signaling by conditional knockout of RBPJ (recombining binding protein suppressor of hairless, also known as CBF1), one of the key transcription cofactors for NICD1, completely abrogated the* Kras* ^*G12V*^-induced lung adenocarcinoma formation in* LSL-Kras* ^*G12V*^ *; Rbpj* ^*lox*/*lox*^ mice [25]. Similarly, pharmacological inhibition of NOTCH by treating the* LSL-Kras* ^*G12V*^ mice with *γ*-secretase inhibitor (LSN-411575) also significantly decreased the overall lung tumor burdens [25]. Interestingly, *γ*-secretase inhibitor treatment (with both LSN-411575 and DAPT) increased the expression of DUSP1 and inactivated ERK1/2 MAP kinases [25], suggesting a critical role for the DUSP1/ERK axis as a mediator of activated NOTCH1 in mutant* KRAS*-dependent lung tumorigenesis. Moreover, inactivation of NOTCH1 signaling by expressing a dominant negative form of* Mastermind-like-1* (DN*Maml1*) (without the necessary coding sequences for the recruitment of transcription coactivators [26]), another important transcription cofactor for NICD1, also significantly inhibited the mutant* KRAS*-dependent lung adenocarcinoma formation in the* CC10-CreER; K-RasG12D; Rosa26-DNMaml1-GFP* mice [27]. Notably, these mice also developed squamous hyperplasia in the alveoli [27], suggesting that inactivation of NOTCH1 signaling by dominant negative Maml1 may have distinct effects on the malignant transformation of different types of lung epithelial cells and the development of subtypes of lung tumors. Collectively, these mouse model studies clearly show a prooncogenic role for NOTCH1 in lung adenocarcinoma development within a* KRAS*-mutated cellular context. In future studies, it would be interesting to examine whether NOTCH1 has distinct or differential roles in lung adenocarcinoma cells that harbor other gene mutations, especially the mutations that do not coexist with* KRAS* mutations in lung cancer cells, for instance,* EGFR* mutations [28, 29].

## 3. Emerging Roles of NOTCH1 in Metastasis

### 3.1. Role of NOTCH1 in NSCLC Metastasis

**Figure 2:**
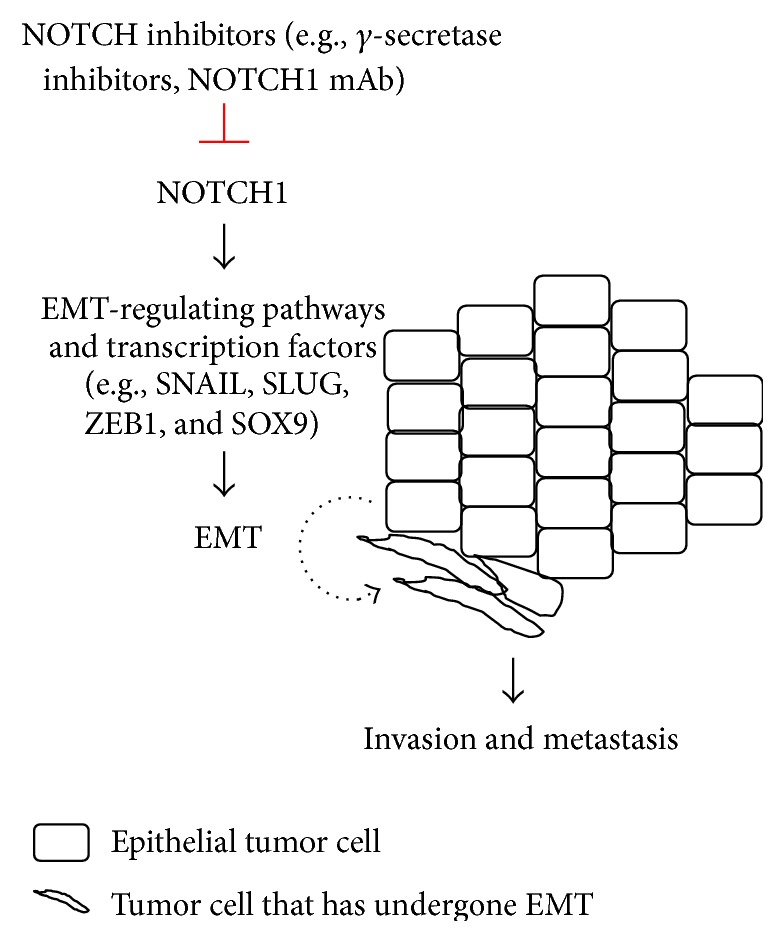
Hypothetical role of NOTCH1 in EMT and metastasis. Elevated NOTCH1 activity in epithelial tumor cells may promote EMT through EMT-regulating pathways and transcription factors, such as SNAIL, SLUG, ZEB1, and SOX9. Tumor cells that have undergone EMT display mesenchymal morphology and acquire enhanced invasiveness and metastatic potential. Inhibition of NOTCH1 by NOTCH inhibitors, including *γ*-secretase inhibitors and NOTCH1 monoclonal antibodies (mAb), may inhibit or reverse EMT and may be useful for treating metastatic cancers.

An increasing number of studies have shown that NOTCH1 plays a critical role in epithelial-mesenchymal transition (EMT), an early step in cancer metastasis (reviewed in [43–46]). NOTCH1 may regulate EMT through both indirect mechanisms (e.g., cross talk with other EMT-regulating pathways, such as the TGF-*β* signaling pathway) and direct regulation of several transcription factors that drive EMT (Figure 2). For instance, NOTCH1 was shown to directly bind to and activate the promoter of SLUG, an E-box-binding transcription factor that drives EMT by repressing epithelial and polarity genes, including E-cadherin [47]. Inhibition of NOTCH1 may not only induce mesenchymal-epithelial transition (MET, namely, the reverse process of EMT), but also suppress the invasion and metastasis of various types of cancer cells [43–46], suggesting that NOTCH1 inhibitors may be useful for treating patients with metastatic cancers, such as advanced stage NSCLC, for which metastasis is the main cause of patient death.

In a mouse model of metastatic lung adenocarcinoma that coexpresses mutant* KRAS* (*KRAS* ^*G12D*^) and* p53* (*p53* ^*R172H*Δ*G*^), we have shown that the NOTCH ligand Jagged2 was highly expressed by CD133-positive prometastatic lung adenocarcinoma cells [31]. Knockdown of Jagged2 by expressing shRNAs inhibited the activation of NOTCH1, suppressed epithelial-mesenchymal transition (EMT) and invasion, and abrogated metastasis in a syngeneic tumor metastasis model using lung adenocarcinoma cell lines derived from the* KRAS* ^*G12D*^;* p53* ^*R172H*Δ*G*^ mice [31]. Consistently, a recent report has shown that the expression of Galectin-1, a glycan binding protein overexpressed in lung cancer, increased the expression of both Jagged2 and NOTCH1 and promoted Lewis lung carcinoma metastasis [32]. Expression of Galectin-1 in lung adenocarcinoma cells (CL1-0 and A549) promoted EMT, migration, and invasion; knockdown of Galectin-1 in these cells reversed EMT and inhibited migration and invasion [32]. Knockdown of NOTCH1 significantly suppressed AKT activation, migration, and invasion driven by the Galectin-1, suggesting a role for NOTCH1 as a direct mediator of Galectin-1 in these processes [32]. It was also shown that NOTCH1 can be activated by ADAM10 (A Disintegrin and metalloproteinase domain-containing protein 10), a sheddase with *α*-secretase activity, and promoted migration and invasion of the A549 lung adenocarcinoma cells [33]. Expression of NICD1 in various human lung cancer cell lines (A549, H1650, and H596) induced EMT and destroyed adherens junctions by increasing the Snail family of E-box-binding transcription repressors, including SNAI1 and SLUG, which in turn repressed the expression of E-cadherin and *β*-catenin [34]. In both human lung adenocarcinoma lines (H1838 and H322 cells) and immortalized lung epithelial cells (BEAS2B), the expression of NICD1 increased SOX9 (sex determining region Y-box 9), a transcription factor promoting EMT, migration, and invasion of lung adenocarcinoma cells [35]. NICD1 was found to directly bind to the* SOX9* gene promoter elements and to activate its transcription, demonstrating a molecular mechanism by which NOTCH1 drives EMT and invasion in lung adenocarcinoma cells [35]. Collectively, these findings suggest that targeting NOTCH1 may be a useful strategy for treating or preventing NSCLC metastasis.

### 3.2. Role of NOTCH1 in SCLC Metastasis

The role of NOTCH1 in the metastasis of SCLC remains poorly explored. In a sharp contrast to the above-mentioned findings from NSCLC cells, a recent report has shown that knockdown of NOTCH1 in SCLC cell lines (H69AR and SBC3) promoted EMT and invasion, and forced expression of NICD1 in the H69 SCLC cells had an opposite effect [30], suggesting that inhibition of NOTCH1 may further promote the invasion and metastasis of SCLC. These results also suggest that targeting NOTCH1 in SCLC and NSCLC may have distinct clinical outcomes. Nevertheless, most of the above-mentioned studies (for both SCLC and NSCLC) utilized* in vitro* assays to address the role of NOTCH1 in metastasis, and future studies that assess the* in vivo* impact of NOTCH1 on spontaneous metastasis are desirable.

## 4. Clinical Significance of NOTCH1

### 4.1. The Prognostic Value of NOTCH1

Correlative studies based on immunohistochemical (IHC) staining of lung cancer tissues for NOTCH1 expression have led to contradictory conclusions (Table 1). Several studies have shown that NSCLC tissues have significantly higher NOTCH1 expression compared to normal lung tissues, and the expression level of NOTCH1 positively correlates with disease progression, metastasis, and worse patient survival. For instance, positive NOTCH1 staining was found in about half (43.9%) of the NSCLC tissues (*n* = 305, consisting of 210 squamous carcinomas and 95 adenocarcinomas), whereas only 15% of the normal lung tissues (*n* = 80) were positive for NOTCH1 staining [36]. Furthermore, the NOTCH1 expression level in the tumors positively correlated with not only their TNM stages (the tumor/node/metastasis cancer staging system) and lymph node metastasis but also shorter patients' postoperative survival time [36]. In line with these results, another study showed that the positive NOTCH1 IHC staining was found in 81.5% of the NSCLC tissues (*n* = 65, consisting of 38 squamous cell carcinomas and 27 adenocarcinomas), and the expression level of NOTCH1 positively correlated with both disease stages and lymph node metastasis [37]. However, the clinical significance of NOTCH1 in each of the two NSCLC subtypes in the above studies, including squamous cell carcinoma and adenocarcinoma, was unclear. A recent study [38] showed that higher NOTCH1 was expressed in 44% of adenocarcinomas (*n* = 113), 35% of large cell carcinomas (*n* = 31), and 26% of squamous cell carcinomas (*n* = 191). NOTCH1 expression level was found to be an independent prognostic factor that predicted worse survival of lung adenocarcinomas (*n* = 111), but not squamous cell carcinomas [38]. Although it was not an independent prognostic factor for squamous cell carcinoma, the combination of NOTCH1 and VEGF-A (vascular endothelial growth factor A) expression levels did predict worse patient survival (*n* = 164) [38]. These results are consistent with a recent meta-analysis of 19 studies (including a total of 3663 NSCLC patients) showing that NOTCH1 expression (quantitated by either RT-PCR or IHC scoring) positively correlated with both lymph node metastasis and high TNM stages [39]. Though not statistically significant in either lung adenocarcinoma or squamous cell carcinoma alone, higher NOTCH1 level predicted overall poorer patient survival, suggesting that targeting NOTCH1 may be useful for a subset of NSCLC patients [39].

### 4.2. Gain-of-Function Mutations of NOTCH1

Recent sequencing of the entire C-terminal region of* NOTCH1*'s coding sequence (CDS), including exons 26, 27, and 34, for 49 NSCLC tissues (consisting of 15 squamous cell carcinomas and 34 adenocarcinomas) identified four heterozygous mutations of* NOTCH1* from a total of six tissues (3 squamous cell carcinomas and 3 adenocarcinomas) [40]. One of these mutations,* V2444fs*, was a recurring mutation detected in three tissues (2 squamous cell adenocarcinomas and 1 adenocarcinoma); each of the other three mutations was identified in a single tissue, either a squamous cell carcinoma (*S2275fs*) or an adenocarcinoma (*R2328W* and* D1643H*) [40]. Individual ectopic expression of all these NOTCH1 mutants in HeLa cells activated a CBF1-luciferase reporter, which can be inhibited by treatment with the *γ*-secretase inhibitors MRK-003 and DAPT, suggesting that these NOTCH1 mutations are gain-of-function mutations [40]. Consistently, the activated NOTCH1 level positively correlated with poorer survival of NSCLC patients (*n* = 420, consisting of 176 squamous cell carcinomas and 244 adenocarcinomas) without p53 mutations (48.9% of the entire cohort of 420 patients), suggesting that targeting NOTCH1 may be a useful strategy for treatment of certain lung cancer patients, especially those with gain-of-function NOTCH1 mutations and/or without p53 mutations [40].

### 4.3. Contradictory Findings

On the contrary to the above-mentioned results, Huang et al. showed that lung adenocarcinoma cell lines (SPC-A1, A549, and H1299) expressed lower NOTCH1 than a nontumorigenic lung epithelial cell line did (16HBE cells), and positive NOTCH1 staining was detected in both human lung adenocarcinoma tissues and their adjacent normal alveolar and bronchial lung epithelial tissues [41]. IHC staining results showed that NOTCH1 was primarily expressed on the membrane and/or in the cytosol of the tumor cells, and a significantly higher percentage of stage I tumors (25 out of 45 tissues) were positive for NOTCH1 staining compared to tumors at later clinical stages (stages II–IV; 11 out of 56 tissues were positive) [41]. The expression level of NOTCH1 negatively correlated with lymph node metastasis and predicted longer patient survival (*n* = 101) [41]. Interestingly, this work also showed that the solid predominant adenocarcinoma (SPA) tissues had a much lower ratio of NOTCH1 positive staining (3 out of 25 were positive) compared to the other two pathological subtypes of adenocarcinomas (26 out of 64 were positive), including the acinar predominant adenocarcinomas (APA) and papillary predominant adenocarcinomas (PPA), suggesting that NOTCH1 may be a useful biomarker for differentiating histological subtypes of lung adenocarcinoma [41]. Partly consistent with these findings, Nguyen et al. showed that 50% of the NSCLC tissues (*n* = 58, consisting of 35 squamous cell carcinomas, 19 adenocarcinomas, and 4 undifferentiated/large cell carcinomas) were positive for NOTCH1 staining, and NOTCH1 was predominantly localized to the membrane and cytosol of the tumor cells [42]. Nuclear NICD1 expression was only detected in a small proportion of the tissues (12%) [42]. While NOTCH1 expression negatively correlated with both clinical stages and lymph node metastasis, NICD1 expression did not significantly correlate with any of them [42]. In addition, the whole exome sequencing of 40 TCGA (the Cancer Genome Atlas, http://cancergenome.nih.gov/) lung squamous cell carcinomas identified four nonrecurring* NOTCH1* mutations from three tissues, including a splice site mutation and three missense mutations,* I1440T*,* C429S*, and* R353C* (the last two were from the same tissue) [48]. Though not experimentally validated, two of these mutations, including the splice site mutation and the* R353C* mutation, were predicted as potentially damaging mutations that may inactivate NOTCH1 activity based on the analysis of their potential structural effects [48].

## 5. Conclusion

Overall, our current understanding of the role of NOTCH1 in lung cancer is still incomplete. A limited number of studies suggest that NOTCH1 may act as a tumor suppressor in SCLC. For NSCLC, contradictory findings have been reported (summarized in Table 1). The biological and pathological basis for such divergence is unclear. The role of NOTCH1 may be more complicated than previously expected and may highly depend on the genetic, cell, and pathological contexts. This may also partly explain why controversial results were reported in correlative studies using clinical samples, as many factors, such as the demographic and clinical characteristics of the tissues, sample size, gene mutation status, and prior treatments, may collectively determine the results of these studies. These findings also warrant future research that examines whether targeting NOTCH1 should be used as personalized strategy for a subset of lung cancer patients, for instance, the patients with gain-of-function NOTCH1 mutations.

## Figures and Tables

**Table 1:** Key references for the role of NOTCH1 in lung cancer.

References	Subtype	Key findings	Potential role of NOTCH1
[10, 11]	SCLC	NOTCH1 inhibited cell proliferation and neuroendocrine marker expression.	Tumor suppressive

[12, 13]	SCLC	NOTCH1 induced growth inhibition and cell cycle arrest.	Tumor suppressive

[30]	SCLC	NOTCH1 inhibited EMT and invasion.	Tumor suppressive

[14]	NSCLC	Knockdown of NOTCH1 inhibited cell growth; NICD1 promoted cell growth in the presence of EGF.	Oncogenic

[15, 16]	NSCLC	Hypoxia-induced HIF1*α* activated NOTCH1 to promote cell growth; the *γ*-secretase inhibitor MRK-003 induced cell apoptosis under the condition of hypoxia.	Oncogenic

[23–27]	NSCLC	Inactivation of NOTCH1 or its mediators in mouse models of NSCLC abrogated tumorigenesis.	Oncogenic

[31]	NSCLC	Inactivation of the NOTCH ligand Jagged2 inhibited EMT and metastasis.	Oncogenic

[32]	NSCLC	Galectin-1 increased Jagged2 and NOTCH1 to promote metastasis.	Oncogenic

[33]	NSCLC	ADAM10 activated NOTCH1 to promote invasion.	Oncogenic

[34]	NSCLC	NICD1 induced EMT and destroyed adherens junctions.	Oncogenic

[35]	NSCLC	NICD1 transcriptionally activated SOX9 to drive EMT and invasion.	Oncogenic

[36–39]	NSCLC	Higher NOTCH1 correlated with disease progression, metastasis, and poorer prognosis.	Oncogenic

[40]	NSCLC	Gain-of-function NOTCH1 mutations are identified in a subset of patients; activated NOTCH1 activity correlated with poorer survival of NSCLC patients without p53 mutations.	Oncogenic

[17]	NSCLC	NICD1 inhibited cell and xenograft tumor growth.	Tumor suppressive

[18]	NSCLC	NOTCH1 mediated Z-Isochaihulactone-induced growth inhibition.	Tumor suppressive

[19]	NSCLC	Endothelial DLL4 activated tumor cell NOTCH1 to inhibit growth.	Tumor suppressive

[41]	NSCLC	NOTCH1 expression negatively correlated with metastasis and predicted better survival.	Tumor suppressive

[42]	NSCLC	NICD1 was only detected in a small proportion of patient tissues and had no prognostic value.	
